# NetCleave: an open-source algorithm for predicting C-terminal antigen processing for MHC-I and MHC-II

**DOI:** 10.1038/s41598-021-92632-y

**Published:** 2021-06-23

**Authors:** Pep Amengual-Rigo, Victor Guallar

**Affiliations:** 1 Barcelona Supercomputing Center (BSC), 08034 Barcelona, Spain; 2 ICREA: Institució Catalana de Recerca I Estudis Avançats, Passeig Lluís Companys 23, 08010 Barcelona, Spain

**Keywords:** Immunotherapy, Machine learning, Cancer therapy, MHC

## Abstract

Antigens presented on the cell surface have been subjected to multiple biological processes. Among them, C-terminal antigen processing constitutes one of the main bottlenecks of the peptide presentation pathways, as it delimits the peptidome that will be subjected downstream. Here, we present NetCleave, an open-source and retrainable algorithm for the prediction of the C-terminal antigen processing for both MHC-I and MHC-II pathways. NetCleave architecture consists of a neural network trained on 46 different physicochemical descriptors of the cleavage site amino acids. Our results demonstrate that prediction of C-terminal antigen processing achieves high accuracy on MHC-I (AUC of 0.91), while it remains challenging for MHC-II (AUC of 0.66). Moreover, we evaluated the performance of NetCleave and other prediction tools for the evaluation of four independent immunogenicity datasets (H2-Db, H2-Kb, HLA-A*02:01 and HLA-B:07:02). Overall, we demonstrate that NetCleave stands out as one of the best algorithms for the prediction of C-terminal processing, and we provide one of the first evidence that C-terminal processing predictions may help in the discovery of immunogenic peptides.

## Introduction

Adaptive immune system has evolved to locate, degrade and expose antigen sources to the T-cell repertoire, aiming to eliminate potential threats. This herculean task is accomplished by the antigen presentation pathways, which are composed by a complex network of specialized cells, proteolytic enzymes, peptide recognition and transportation, and protein–protein binding events. There are two major antigen presentation pathways and their end-point process, the peptide binding to the Major Histocompatibility Complex (MHC), names them: class I and class II pathways.

During the past decades, there has been an interest in deciphering the molecular basis of the presentation pathways for developing predictive models. In this context, peptide binding predictions have classically attracted substantially more attention than any other molecular process^1–7^. Without lack of controversy in the field, peptide binding affinity has been suggested to be an inefficient metric for the determination of its immunogenic potential^8^. Recently, it has been described that other determinations play important roles for the enrichment of immunogenic peptides, including for instance expression levels of antigens genes and/or protease cleavage signatures^9^. Hence, for the development of an efficient immunogenic response, the antigen source gene must be expressed, and the antigen must be processed and bound to the MHC, among other critical processes.

Independently of the presentation pathway, the initial bottleneck step for the generation of antigens consists of the source protein proteolysis into smaller peptides. Each pathway generates its specific peptidome by means of different proteolytic enzymes. In this regard, class I pathway is mainly fed by the proteasome (and immunoproteasome), while class II is fed by cathepsins. From the computational point of view, the cleavage signatures of the proteasome and cathepsins remain challenging to model. On the one hand, the proteasome shows three different proteolytic activities for hydrophobic, basic and acidic cleavage signatures: chymotrypsin-like, trypsin-like and peptidyl-glutamyl peptide-hydrolyzing (PHGH)-like, respectively^10^. Moreover, proteasome cleavage specificity is modulated by the current immunological status of the host, which may shape the peptidome content^11,12^. On the other site, several cathepsins with different cleavage specificities have been described belonging to serine proteases (cathepsin A and G), aspartic proteases (cathepsin D and E) and cysteine proteases (cathepsin B, C, F, H, K, L, O, S, V, X, and W)^13^. Some of these proteolytic enzymes are poorly characterized, which hampers the development of efficient predictive algorithms for the overall process.

Most of the research on this topic has focused on the prediction of proteasome cleavage signatures, rather than cathepsin ones. Relatively few algorithms can be found for predicting proteasome cleavage, including PAProc^14,15^, MAPPP^16,17^, NetChop^18,19^, iPCPs^20,21^ and the last version of MHCflurry2.0^22^, which includes a new module for peptide processing prediction. Except for MHCflurry2.0, the code of the above stated algorithms is not available to the public and therefore, those methods cannot be retrained by users. In fact, some of those methods were developed almost two decades ago, and during this time, large amounts of data has been released and it is not currently being considered in the predictions. Therefore, having a retrainable and freely accessible algorithm is crucial for the continuity of the research in this field.

Recent advances in the use and applicability of mass spectroscopy (MS) techniques provide a photo finish of both antigen presentation pathways, identifying large amounts of peptides naturally presented on the MHC^23^. To date, 318.203 and 117.781 unique peptides elicited by MS assays are publicly available on the IEDB database for class I and class II receptors, respectively^7^. In this work, we envisioned NetCleave, an open-source and easily retrainable algorithm for the prediction of C-terminal antigen processing. In this sense, NetCleave can be retrained on particular conditions, such as allele or isotype specific models, host or pathogen specific models, and/or particular presenting cell models, among others. Similarly to NetChop3.1, which is the most used algorithm for proteasome prediction, we also envisioned a standard artificial feed-forward neural network with one hidden layer. However, instead of following a one-hot encoding scheme, we feeded our neural network with a set 46 different amino acid descriptors (16 hydrophobic, 17 steric and 15 electronic features) publicly available^24^, as previously used in the literature^25^.

Our results suggest that NetCleave predictions achieve great predictive power towards class I isotypes (AUC ~ 0.92) and modest predictive power towards class II isotypes (AUC ~ 0.66). In this context, the C-terminal region of the class I peptides is crucial for binding since it contains important anchor residues. However, this is not the case for class II peptides, whose C-terminal region typically falls outside of the receptor binding groove. Taking everything together and considering the large amount of cathepsins that have been described with different cleavage specificities, the drop in predictive performance for class II was expected compared to class I. Moreover, we compared the performance of NetCleave with other algorithms for predicting a set of immunogenic and non-immunogenic peptides from the four most-well characterized alleles on the IEDB: two mice alleles (H2-Db and H2-Kb) and two human alleles (HLA-A*02:01 and HLA-B*07:02). Here, we demonstrate that NetCleave is currently one of the best algorithms for the prediction of C-terminal processing and, thus, a good candidate to be used in combination with other prediction tools (such as MHC binding, etc.) for the identification of immunogenic peptides.

## Methods

### Data generation

Eluted peptides from MS determinations were obtained from the IEDB database^26^. This database contains detailed information for each peptide, including the reporting literature reference, epitope information, host and pathogen data, the experimental technique used, presenting cell type and the MHC class associated receptor. Hence, NetCleave framework was built aiming to allow personalized peptide selections based on multiple conditions, such as for instance: human peptides associated with cancer and presented on the MHC class I alleles, or viral peptides presented on the MHC class II alleles of the dendritic cells.

**Figure 1:**
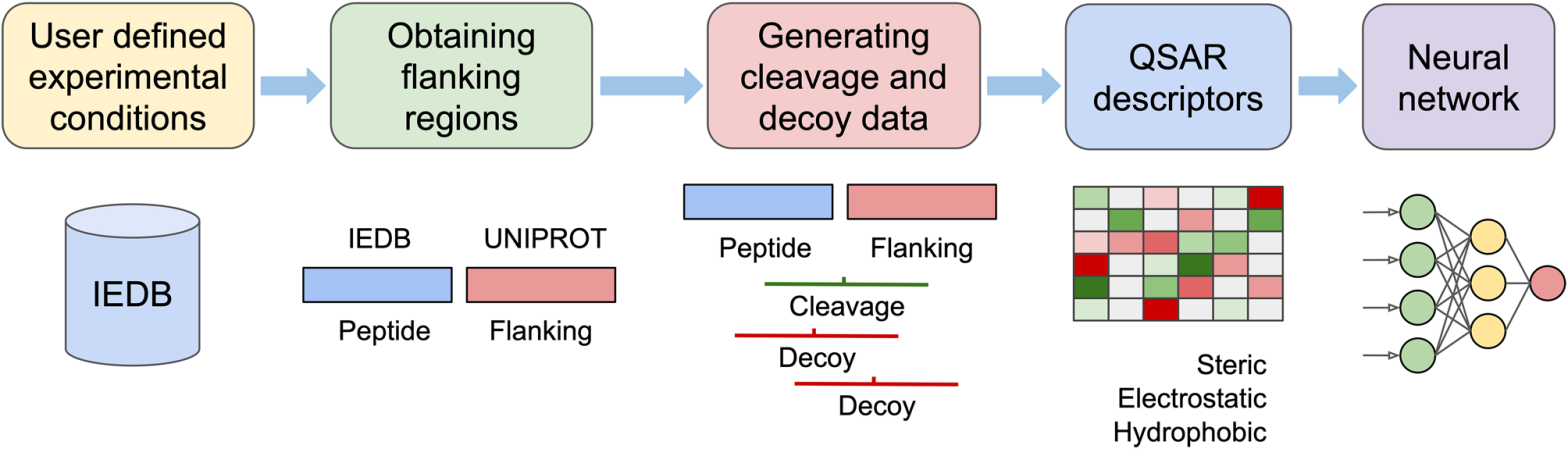
Overview of the NetCleave data preparation framework. Peptides reported by defined experimental conditions are automatically selected from the IEDB, and their C-terminal flanking residues are retrieved from UniParc/UniProt. Cleavage and decoy data of seven residue length are generated and encoded by 46 QSAR descriptors consisting of steric, electrostatic and hydrophobic properties.

An overview of the NetCleave data generation process is illustrated in Fig. 1. After the definition of the experimental conditions, NetCleave collects from the IEDB all unique peptides coming from the same source protein (by UniProt identifier). Then, flanking C-terminal sequences are retrieved from UniParc/UniProt^27^. As negative samples are not detected in MS assays, we followed a standard decoy data generation procedure assuming that the cleavage probability for observed cleavage sites (at position P1–P1ʹ) is higher than the neighbouring ones (at positions P2–P1 and P1ʹ–P2ʹ). Hence, two decoy samples adjacent to the cleavage sites are used as negative samples.

NetCleave uses a short sequence of seven residues to generate predictions: four residues placed before and three after the cleavage site. Each residue is encoded with 46 amino acid descriptors (describing steric, electrostatic and hydrophobic properties)^24^, resulting in a total amount of 336 descriptors for each short sequence. To feed the NetCleave neural network, the observed cleavage sites from the MS data are labeled as 1 and decoy samples are labeled as 0.

### Neural network architecture

**Figure 2:**
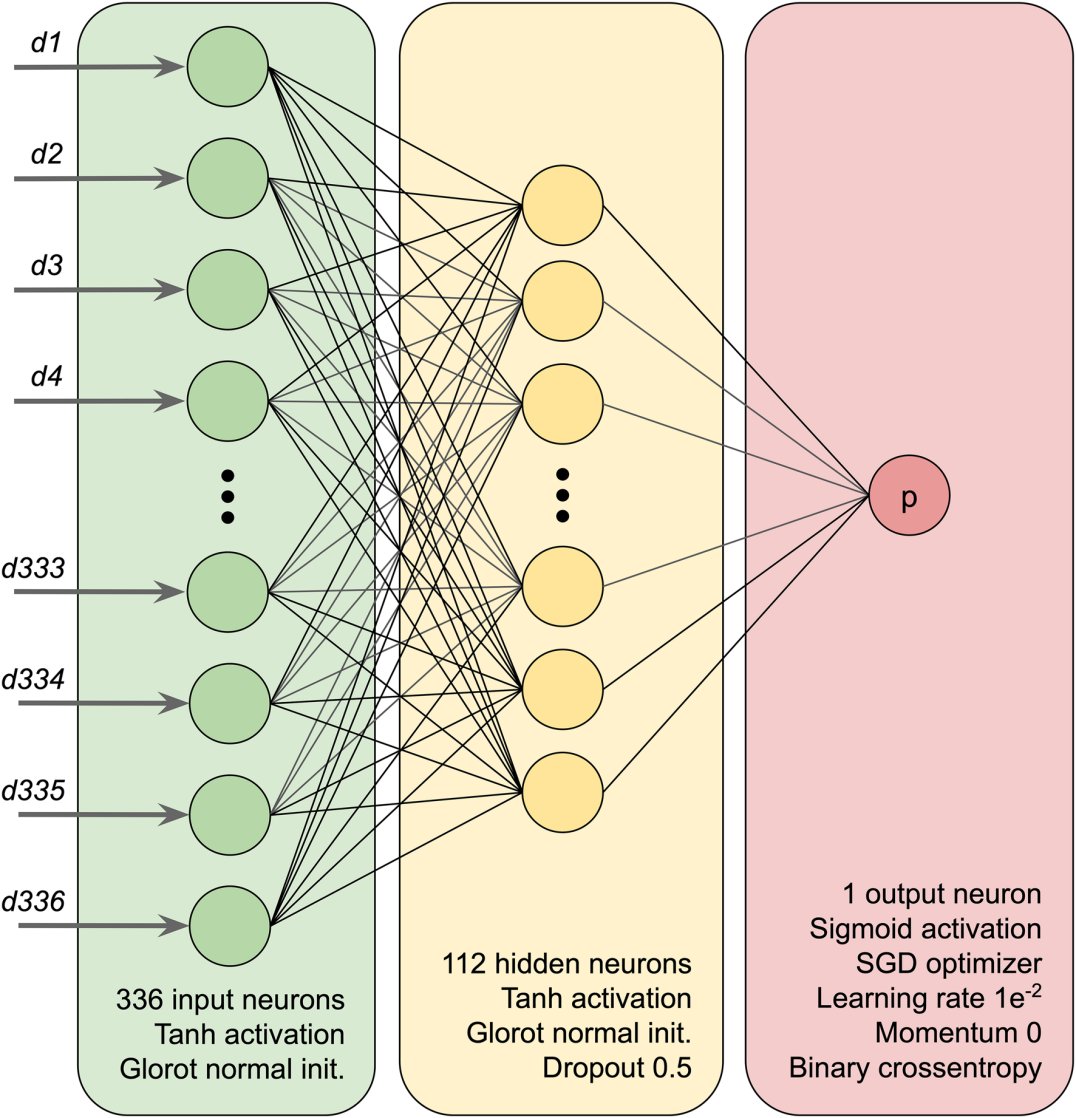
NetCleave neural network architecture. For the input layer (green), an input neuron is defined for each of the 336 physicochemical descriptors, using a Tanh activation and a Glorot normal initialization function. One third of the input neurons is used on the hidden layer (yellow), which takes advantage of the same activation and initialization functions of the input layer. A dropout of 0.5 was used to prevent overfitting. For the output layer (red), a single output neuron is used following a sigmoid activation function. Model compilation consisted of using the SGD optimizer with no momentum, a learning rate of 1e^−2^ and a binary cross entropy loss.

An overview of the NetCleave neural network architecture is illustrated in Fig. 2. Amino acid descriptors are standardized by removing the mean and scaling to standard deviation. Each descriptor is used to feed a neuron of the input layer, using a hyperbolic tangent (Tanh) activation function and Glorot normal initialization to set the initial random weights. One third of the input neurons are used on the single hidden layer, which is activated by Tanh function and initialized by Glorot normal function. A dropout of 0.5 was defined to prevent overfitting. A single neuron is used in the output layer, which is activated by a sigmoid function. Hence, NetCleave score represents the cleavage probability between the range of 0 and 1, where the former and the latter represent the minimum and the maximum cleavage probabilities, respectively. For compiling the model, we used a stochastic gradient descent (SGD) optimizer with no momentum. A learning rate of 1e^−2^ and a binary cross entropy loss were defined. During the construction of the models, 60%, 30% and 10% of the data is splitted for training, validation and testing groups.

Several statistical metrics were used for the evaluation of the model performance: binary accuracy, precision (also known as positive predictive value, PPV, Eq. (1)), recall (also known as true positive rate, TPR, Eq. (2)), Matthew’s coefficient correlation (MCC, Eq. (3)) and the area under the curve (AUC). A classical threshold of 0.5 was used to determine if the cleavage site is predicted to be processed or not.

1 $$PPV = \frac{{TP}}{{TP + FP}}$$

2 $$TPR = \frac{{TP}}{{TP + FN}}$$

3 $$MCC = \frac{{\left( {TPxTN} \right) - \left( {FPxFN} \right)}}{{\sqrt {\left( {TP + FP} \right)\left( {TP + FN} \right)\left( {TN + FP} \right)\left( {TN + FN} \right)} }}$$

### Evaluation of immunogenic datasets

NetCleave performance was compared with iPCPs, NetChop3.1, and MHCflurry2.0 for evaluating immunogenic data for the four most well represented alleles of the IEDB, consisting of two mice alleles (H2-Db and H2-Kb), and two human alleles (HLA-A*02:01 and HLA-B*07:02). Most of the immunogenicity samples were measured using the ELISPOT technique. Hence, aiming to reduce bias among different techniques, only peptides determined by this method were evaluated. Moreover, a qualitative immunogenicity value is provided for each database entry, consisting of “Negative”, “Positive”, “Positive-Low”, “Positive-Intermediate” and “Positive-High”. We simplified this complex categorization scheme into a simple binary classification strategy: negative and positive, where the latter is constituted by any positive determination independently of the immunogenicity strength. Moreover, some peptides have been reported in different assays to belong to both negative and positive immunogenicity groups. Therefore, peptides not homogeneously described to be either negative or positive immunogenic for the same allele were discarded. After this data curation process, a set of 98–1273, 157–1648, 233–1030 and 84–421 immunogenic/non-immunogenic peptides were selected for H2-Db, H2-Kb, HLA-A*02:01 and HLA-B*07:02, respectively. Note the imbalance of these data sets, that could affect the machine learning procedure. We expect that future data will address this point and have designed NetCleave for an easy retraining.

A threshold of 0.5 was used to determine if the cleavage site is predicted to be processed or not for all evaluated algorithms. From the resulting predictions, TPR, PPV and the area under the curve (AUC) were computed to highlight the performance of all methods on immunogenic datasets.

## Results

### NetCleave performance on cleavage and decoy samples

NetCleave performance was assessed on several models aiming to expose current challenges on the prediction of C-terminal antigen processing. Here, we report performance metrics on eight different groups, consisting of six isotype models (HLA-A, -B, -C, -DP, -DQ and -DR) and two class models (HLA class I and II).

**Figure 3:**
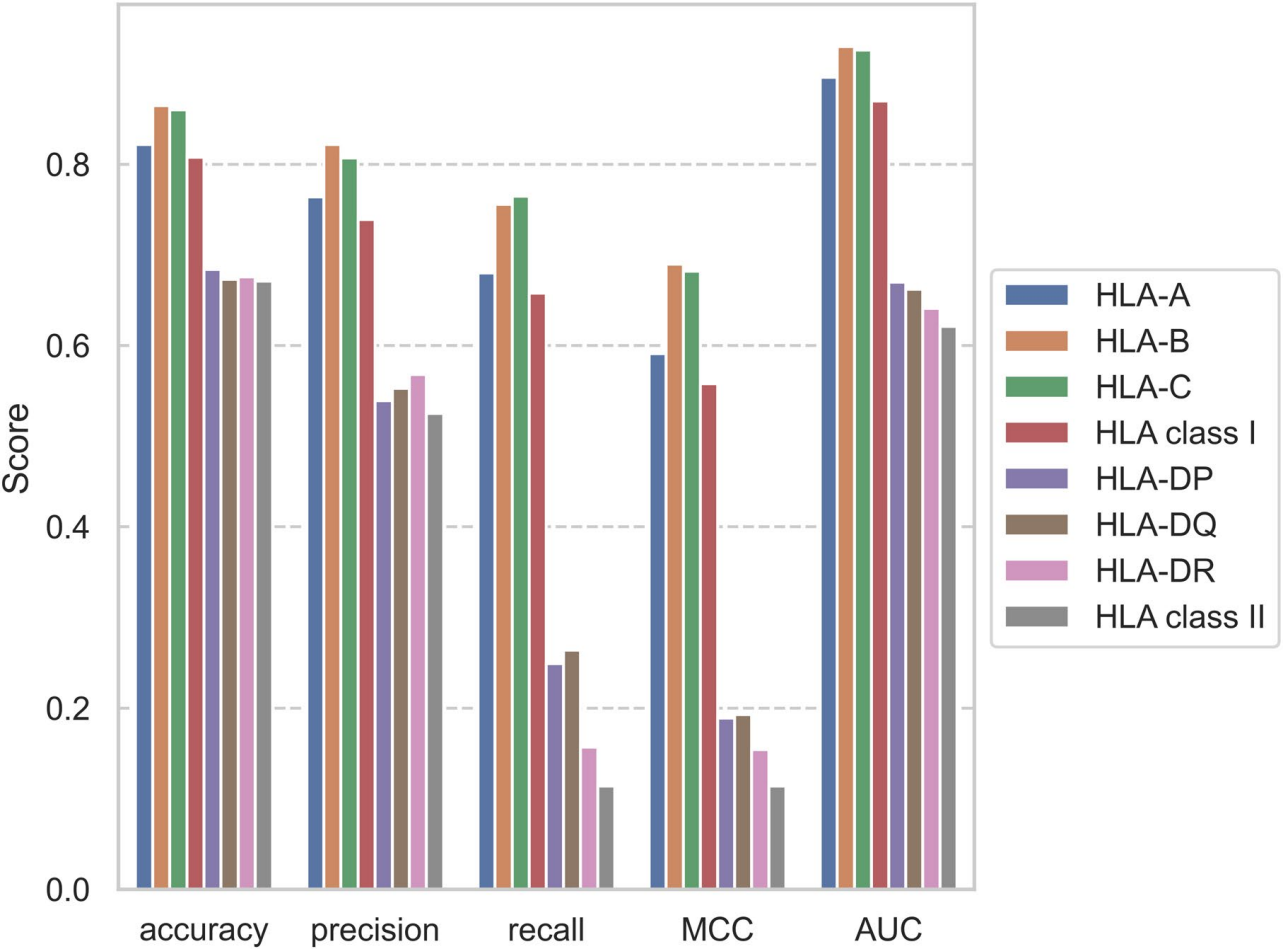
Statistical scores of the NetCleave models on six human allele isotypes (HLA-A, -B, -C, -DP, -DQ and -DR) and two human allele classes (HLA class I and II), including accuracy, precision, recall, MCC and AUC. A threshold of 0.5 was defined to compute the classification statistic scores.

As can be observed in Table S1–S3, no substantial differences on the statistical scores have been found between the training, validation and test sets for each group. This indicates that NetCleave does not show bias towards the training data, and that the expected predictive power on new cases should be similar to the reported ones. Performance on the test sets is illustrated in Fig. 3. Here, accuracy, precision, recall, MCC and AUC scores are represented for isotype and class groups. As can be observed, prediction of isotype class II cleavage sites (AUC mean of 0.66) is substantially more challenging than class I (AUC mean of 0.91). The same behaviour is also true when comparing class groups, where AUC values for class I and class II are 0.87 and 0.62, respectively. Moreover, a minor drop in classification and correlation performance can be observed for a generalized model (class groups) compared to more specific models (isotype groups).

### NetCleave performance on immunogenicity datasets

A set of non-redundant immunogenic and non-immunogenic antigens for two mice (H2-Db and H2-Kb) and two human alleles (HLA-A*02:01 and HLA-B*07:02) were evaluated by NetCleave, iPCPS, NetChop3.1 and MHCflurry2.0. Two MHCflurry2.0 scores were evaluated: MHCflurry2.0 peptide processing and MHCflurry2.0 antigen presentation. The former represents a score for peptide processing, while the latter considers both peptide processing and binding affinity predictions into a single score. In this benchmark, we followed the logical assumption that the C-terminal region of an immunogenic peptide must be efficiently processed in vivo, and therefore it should be associated with high processing probabilities (and NetCleave values). However, this is not necessarily true for non-immunogenic peptides: the reason behind the lack of immunogenicity for a subset of non-immunogenic peptides could be explained by insufficient C-terminal processing (lower processing probabilities and NetCleave values), among others. Hence, independently of the correlation power with immunogenicity (if any), a C-terminal processing algorithm should achieve high recall (TPR) scores on immunogenicity datasets. We need to emphasize that we do not aim at demonstrating any correlation of NetCleave with immunogenicity, but simply to find discriminators of its efficiency as a C-terminal processing prediction algorithm.

**Figure 4:**
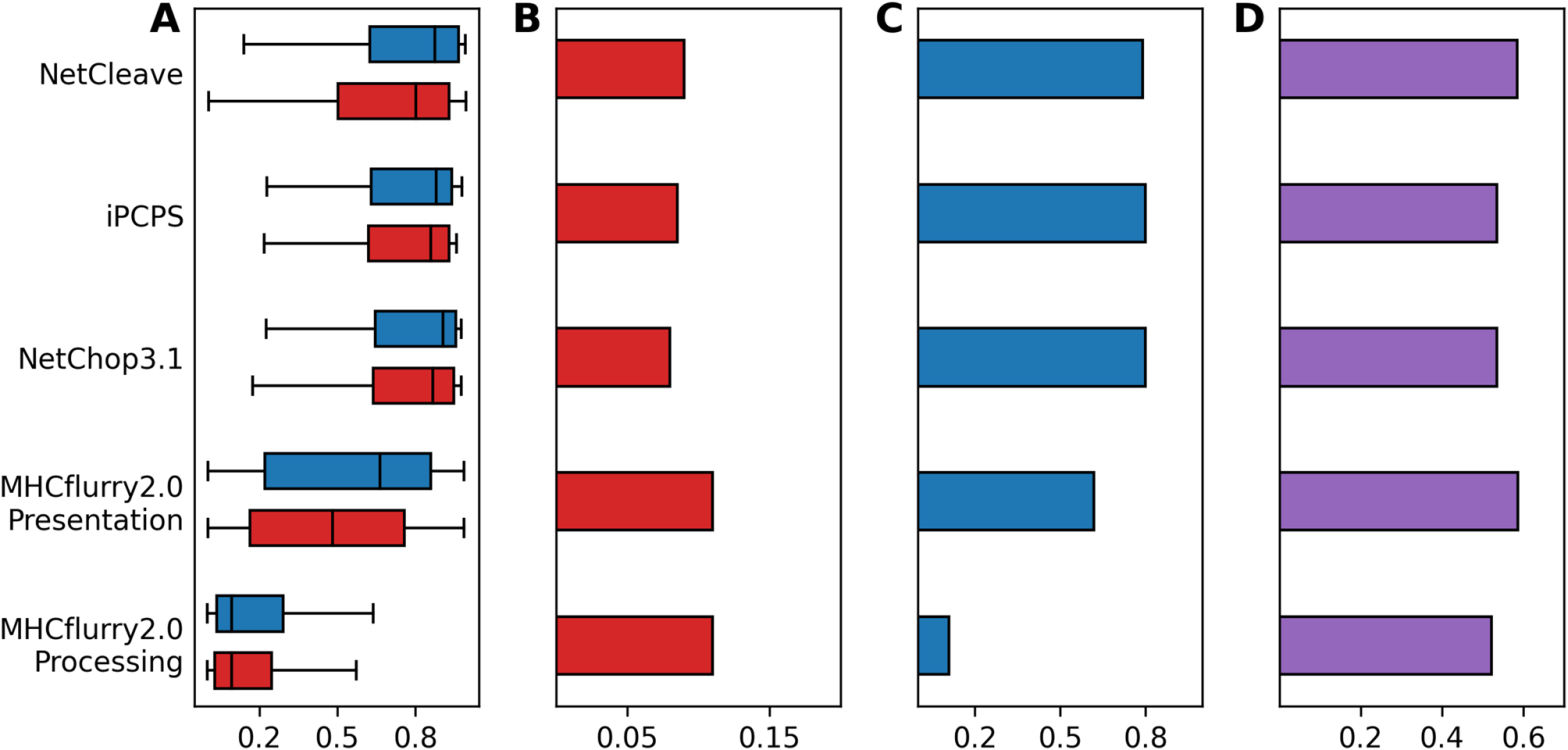
Immunogenicity benchmark of NetCleave, iPCPS, NetChop3.1, MHCflurry2.0 presentation and MHCflurry2.0 processing scores for H2-Kb dataset. (**A**) Prediction distribution for immunogenic (blue) and non-immunogenic peptides (red). (**B**) Precision or positive predictive value (PPV). (**C**) Recall or true positive rate (TPR). (**D**) Area under the curve (AUC) values.

**Figure 5:**
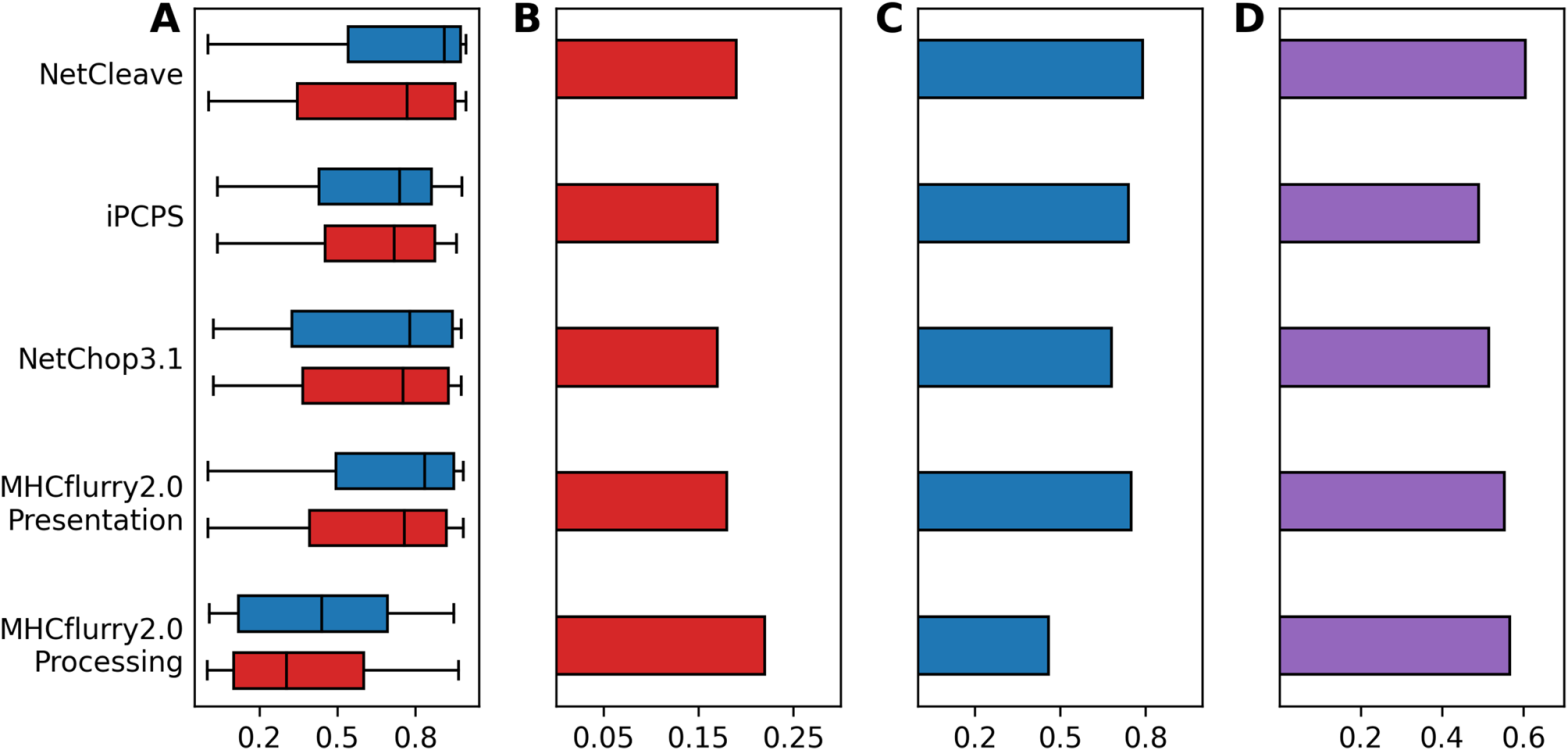
Immunogenicity benchmark of NetCleave, iPCPS, NetChop3.1, MHCflurry2.0 presentation and MHCflurry2.0 processing scores for HLA-B*07:02 dataset. (**A**) Prediction distribution for immunogenic (blue) and non-immunogenic peptides (red). (**B**) Precision or positive predictive value (PPV). (**C**) Recall or true positive rate (TPR). (**D**) Area under the curve (AUC) values.

Immunogenic benchmark results are shown for H2-Kb (Fig. 4), HLA-B*07:02 (Fig. 5), H2-Db (Figure S1) and HLA-A*02:01 (Figure S2). Here, the distribution of the predictive scores of each method, precision (PPV), recall (TPR) and AUC values are shown. As can be observed, NetCleave distribution scores are consistent in all benchmarks. Unlike the other algorithms, NetCleave consistently predicts more than three quarters of the actual immunogenic peptides as processed (TPR > 0.76 in all cases). Moreover, NetCleave AUC values are among the highest in our benchmarks, achieving values of 0.58 (H2-Kb), 0.61 (HLA-B*07:02), 0.61 (HLA-A*02:01) and 0.51 (H2-Db). Regarding the other methods, iPCPS and NetChop3.1 achieve similar distributions for both immunogenic and non-immunogenic groups, which ultimately led to similar statistical scores. In the case of MHCflurry2.0, processing scores of both immunogenic and non-immunogenic groups achieved low predictive values, which is extrapolated into the technique with the lowest TPR. When including binding affinity data into the prediction (MHCflurry2.0 presentation score), distribution probabilities achieved higher scores compared to MHCflurry2.0 processing. However, the TPR of the presentation group tends to achieve lower scores than NetCleave, iPCPS and NetChop3.1.

## Discussion

Recent advances in personalized immunotherapies techniques have attracted the use of computational tools for the prediction of immunogenic antigens or neoantigens for vaccination efforts. In this regard, prediction of immunogenicity is still the major difficulty in the field because of our lack of understanding in the matter. The fact is that a large number of variables could play important roles, which increases the complexity of the overall prediction. Because of that, multiple simplifications have been made aiming to provide insights on the immunogenicity process. The most noticeable assumption is that binding strength to MHC should correlate with immunogenicity. However, this simplification generated much controversy in the field since despite being a prerequisite for the formation of the complex with the T-cell receptor, immunogenicity effects seem to not correlate with binding strength. With the rise of the supercomputational era and the broader accessibility of algorithm resources dedicated to artificial intelligence, larger amounts of variables can be used to train a predictive model. This includes for instance, antigen gene expression, protein expression, antigen processing, antigen transportation, and antigen binding to MHC, among others. Recent studies highlighted that antigen processing data helped to elucidate immunogenic peptides, which motivated the development of NetCleave.

In this regard, C-terminal processing is one of the initial bottlenecks for elucidating which peptides will be presented on the surface of the cell, and therefore, they are potential candidates for the generation of an immune response. In this sense, predictive models can be built on large databases of peptides with binding data to MHC alleles, such as the IEDB. In fact, predictions on peptide processing attracted the attention of researchers around twenty years ago; however, most of the methods have not been updated from their release and cannot take advantage of the enormous data published in recent years.

In this work, we envisioned NetCleave, an open-source platform for the prediction of C-terminal antigen processing that can be easily retrained under the user's needs. We followed the hypothesis that peptides elucidated from MS techniques offer a fair point of view of naturally processed peptides within in vivo systems. Hence, MS peptides should intrinsically provide the rules for an efficient C-terminal processing including protease specificities, peptide transportation mechanisms and peptide binding to MHC peculiarities. Our results demonstrate that C-terminal antigen processing can be accurately predicted for class I alleles (with an AUC ~ 0.91), while it remains challenging for class II alleles (with an AUC ~ 0.66). Historically, predictions on class II alleles have been substantially worse in comparison for class I. The reason behind this effect belongs to the fact that class II alleles are highly promiscuous in peptide length and amino acid composition, compared to class I. Moreover, class II peptides adopt extended conformations on the binding groove allele, where the N-terminal and the C-terminal regions fall outside of the receptor. Hence, there is no biological pressure on this region to be conserved, which also influences the performance of the prediction. Yet another possibility is the fact that substantially more cleavage specificities have been described for proteasome catalytic subunits compared to cathepsins, which also may play an important role from the predictive point of view.

Next, we assessed the performance of NetCleave, iPCPs, NetChop3.1 and MHCflurry2.0 for the cleavage evaluation of four different immunogenic datasets, consisting of two mice alleles (H2-Db and H2-Kb) and two human ones (HLA-A*02:01 and HLA-B*07:02). Our assumption was that peptides reported to be immunogenic must be efficiently processed, and therefore an algorithm for this effect should be able to correctly capture them, giving rise to a high cleavage score. Our results indicate that NetCleave achieves the higher TPR while also showing the higher AUC in the four independent immunogenicity benchmarks.

Overall, we demonstrated that C-terminal antigen processing can be accurately predicted using NetCleave, an open-source code freely available at https://github.com/pepamengual/NetCleave. Since new binding determinations are continuously being deposited on the IEDB, we envisioned a method that could be retrained at any moment by any user. Moreover, specific models can be generated upon user request, which can be extremely useful for the prediction on specialized experimental set-ups.

## Supplementary Information

Supplementary Information.
