# Supplementary material for: NetCleave: an open-source algorithm for predicting C-terminal antigen processing for MHC-I and MHC-II
Source: Sci Rep. 2021 Jun 23;11:13126. doi: 10.1038/s41598-021-92632-y (PMC8222286; doi:10.1038/s41598-021-92632-y)
Supplement: Supplementary file 1 — Supplementary Information. [file 41598_2021_92632_MOESM1_ESM.pdf]

NetCleave: an open-source algorithm for predicting C-terminal antigen processing for MHC-I and MHC-II

Pep Amengual-Rigo[1], Victor Guallar[1,2,*]

[1]Barcelona Supercomputing Center (BSC), 08034 Barcelona, Spain

[2]ICREA: Institució Catalana de Recerca i Estudis Avançats, Passeig Lluís Companys 23, 08010 Barcelona, Spain

[*]Corresponding author

E-mail: victor.guallar@bsc.es

Phone number: +34 93 413 7727

Metrics	HLA-A			HLA-B			HLA-C		
	Training	Validation	Testing	Training	Validation	Testing	Training	Validation	Testing
accuracy	0.836	0.821	0.822	0.870	0.867	0.865	0.866	0.859	0.860
precision	0.786	0.762	0.764	0.832	0.828	0.822	0.814	0.808	0.807
recall	0.696	0.676	0.680	0.768	0.760	0.756	0.772	0.762	0.765
MCC	0.622	0.589	0.591	0.704	0.696	0.690	0.693	0.680	0.682
AUC	0.901	0.896	0.896	0.936	0.931	0.930	0.931	0.925	0.926

Table S1. Statistical determinations for the training, validation and testing groups of NetCleave for each HLA class I isotype (HLA-A, HLA-B and HLA-C). A threshold of 0.5 was used to determine if the cleavage site is predicted to be scored or not, for determining accuracy, precision, recall and MCC statistics. AUC was computed from the ROC curve.

Metrics	HLA-DP			HLA-DQ			HLA-DR		
	Training	Validation	Testing	Training	Validation	Testing	Training	Validation	Testing
accuracy	0.693	0.684	0.684	0.699	0.678	0.673	0.681	0.676	0.676
precision	0.590	0.550	0.539	0.601	0.527	0.553	0.574	0.551	0.568
recall	0.276	0.258	0.249	0.302	0.260	0.264	0.162	0.156	0.157
MCC	0.231	0.198	0.189	0.254	0.186	0.193	0.163	0.146	0.154
AUC	0.695	0.675	0.670	0.700	0.658	0.662	0.660	0.637	0.641

Table S2. Statistical determinations for the training, validation and testing groups of NetCleave for each HLA class II isotype (HLA-DP, HLA-DQ and HLA-DR). A threshold of 0.5 was used to determine if the cleavage site is predicted to be scored or not, for determining accuracy, precision, recall and MCC statistics. AUC was computed from the ROC curve.

Metrics	HLA class I			HLA class II		
	Training	Validation	Testing	Training	Validation	Testing
accuracy	0.812	0.808	0.808	0.679	0.671	0.671
precision	0.744	0.739	0.739	0.577	0.526	0.525
recall	0.662	0.657	0.658	0.124	0.110	0.114
MCC	0.565	0.557	0.558	0.143	0.111	0.114
AUC	0.874	0.870	0.870	0.657	0.618	0.621

Table S3. Statistical determinations for the training, validation and testing groups of NetCleave for each HLA class (HLA class I and HLA class II). A threshold of 0.5 was used to determine if the cleavage site is predicted to be scored or not, for determining accuracy, precision, recall and MCC statistics. AUC was computed from the ROC curve.

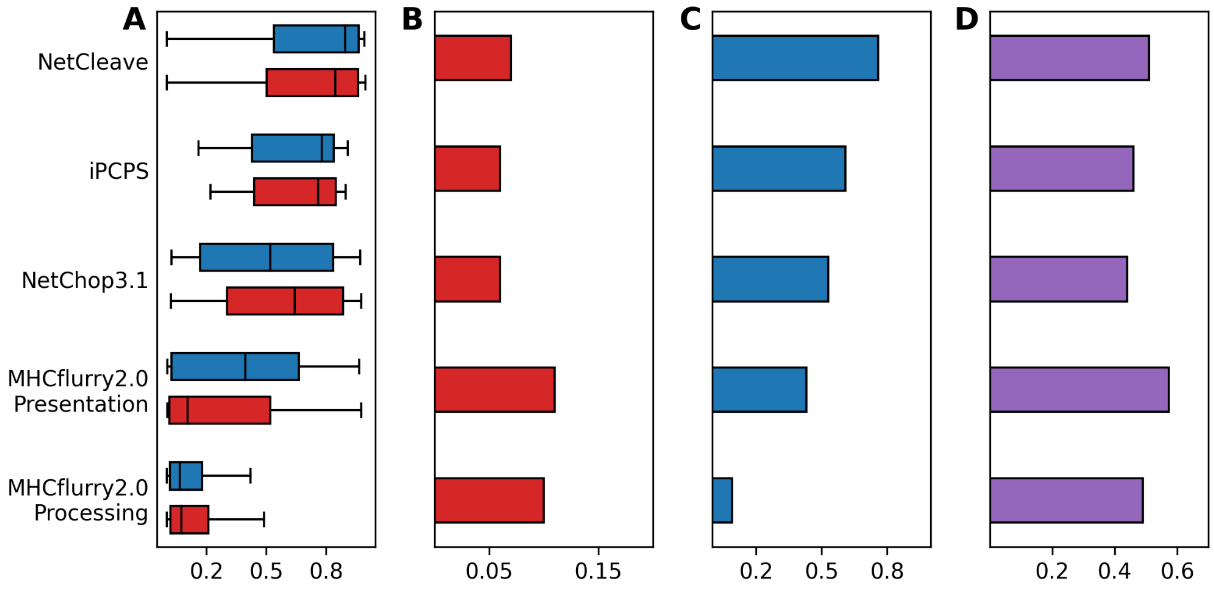

Figure S1. Immunogenicity benchmark of NetCleave, iPCPS, NetChop3.1, MHCflurry2.0 presentation and MHCflurry2.0 processing scores for H2-Db dataset. (A) Prediction distribution for immunogenic (blue) and non-immunogenic peptides (red). (B) Precision or positive predictive value (PPV). (C) Recall or true positive rate (TPR). (D) Area under the curve (AUC) values.

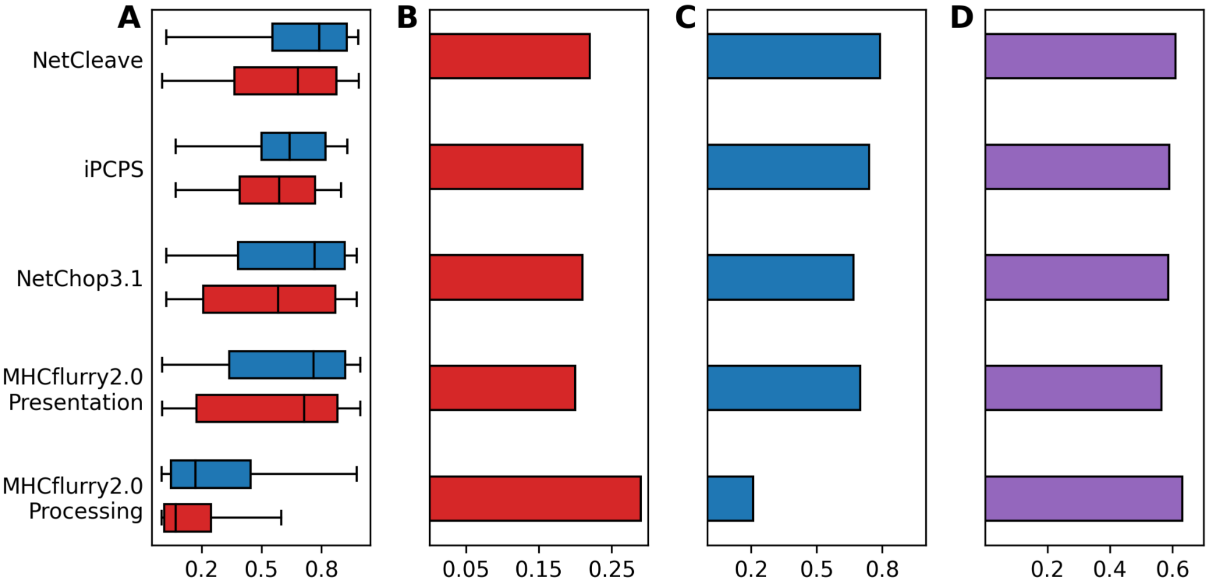

Figure S2. Immunogenicity benchmark of NetCleave, iPCPS, NetChop3.1, MHCflurry2.0 presentation and MHCflurry2.0 processing scores for HLA-A*02:01 dataset. (A) Prediction distribution for immunogenic (blue) and non-immunogenic peptides (red). (B) Precision or positive predictive value (PPV). (C) Recall or true positive rate (TPR). (D) Area under the curve (AUC) values.
